# SARS-CoV-2 Infections among Recent Organ Recipients, March–May 2020, United States

**DOI:** 10.3201/eid2702.204046

**Published:** 2021-02

**Authors:** Jefferson M. Jones, Ian Kracalik, Meenakshi M. Rana, Ann Nguyen, Brian C. Keller, Aaron Mishkin, Charles Hoopes, Thomas Kaleekal, Abhinav Humar, Juan Vilaro, Gene Im, Lou Smith, April Justice, Collette Leaumont, Stephen Lindstrom, Brett Whitaker, Ricardo M. La Hoz, Marian G. Michaels, David Klassen, Wendi Kuhnert, Sridhar V. Basavaraju

**Affiliations:** Centers for Disease Control and Prevention, Atlanta, Georgia, USA (J.M. Jones, I. Kracalik, C. Leaumont, S. Lindstrom, B. Whitaker, W. Kuhnert, S.V. Basavaraju);; Icahn School of Medicine at Mount Sinai, New York, New York, USA (M.M. Rana, G. Im);; University of Chicago, Chicago, Illinois, USA (A. Nguyen);; The Ohio State University College of Medicine, Columbus, Ohio, USA (B.C. Keller);; Temple University, Philadelphia, Pennsylvania, USA (A. Mishkin);; University of Alabama at Birmingham School of Medicine, Birmingham, Alabama, USA (C. Hoopes);; Robert Wood Johnson University, New Brunswick, New Jersey, USA (T. Kaleekal);; University of Pittsburgh Medical Center, Pittsburgh, Pennsylvania (A. Humar, M.G. Michaels);; University of Florida College of Medicine, Gainesville, Florida, USA (J. Vilaro);; New York State Department of Health, Albany, New York, USA (L. Smith);; Ohio Department of Health, Columbus (A. Justice);; University of Texas Southwestern Medical Center, Dallas, Texas, USA (R.M. La Hoz);; United Network of Organ Sharing, Richmond, Virginia, USA (D. Klassen)

**Keywords:** COVID-19, SARS-CoV-2, severe acute respiratory syndrome coronavirus 2, viruses, respiratory infections, zoonoses, coronavirus disease, organ transplantation, transplant recipients, United States, retrospective studies

## Abstract

We conducted public health investigations of 8 organ transplant recipients who tested positive for severe acute respiratory syndrome coronavirus 2 infection. Findings suggest the most likely source of transmission was community or healthcare exposure, not the organ donor. Transplant centers should educate transplant candidates and recipients about infection prevention recommendations.

Although severe acute respiratory syndrome coronavirus 2 (SARS-CoV-2) infection has been reported in organ transplant recipients, it is unclear whether SARS-CoV-2 can be transmitted from organ donors to recipients (*1*) and if transplant recipients are at increased risk for severe illness from coronavirus disease (COVID-19) from SARS-CoV-2 infection compared with immunocompetent patients (*2*). In March 2020, organ procurement organizations (OPOs) and transplant centers in the United States began to report potential donor-derived SARS-CoV-2 transmission to the Organ Procurement and Transplantation Network (OPTN) for investigation by the Disease Transmission Advisory Committee (DTAC). These cases were referred to the Centers for Disease Control and Prevention (CDC), a member of DTAC, to determine if SARS-CoV-2 transmission from a donor had occurred and, if so, to identify the transmission source, and characterize clinical outcomes in the organ recipients.

## The Study

Current OPTN policy requires all US transplant centers and OPOs to report unanticipated potential donor-derived transmission events to the OPTN for investigation by DTAC. CDC coordinates investigations involving pathogens of special interest, including SARS-CoV-2 (Appendix).

For all reported potential donor-derived SARS-CoV-2 transmissions, CDC, OPO, or the transplant center staff reviewed medical records of organ donors and organ recipients. Recipients who initially tested positive for SARS-CoV-2 infection and triggered a notification to the OPTN of a potential donor-derived transmission were referred to as index recipients; recipients who shared a common donor with index recipients were referred to as co-recipients. CDC investigators asked OPO and index recipients’ hospital staff about potential exposures to SARS-CoV-2. Transplant hospital providers monitored organ recipients for symptoms of COVID-19 for >14 days following the transplant. Recipients who developed symptoms and, depending on hospital capacity, some asymptomatic recipients were tested for SARS-CoV-2 infection by a nucleic acid test (NAT). All donor serum were tested for the presence of SARS-CoV-2 RNA. Donor respiratory specimens were tested if available.

During March–May 2020, a total of 8 potential donor-derived transmission events involving 8 deceased donors and 31 recipients were reported to OPTN. Each event was reported because an individual transplant recipient (the index recipient) tested positive for SARS-CoV-2 infection (Table 1; Appendix). For all donors included in this study, the cause of death was determined to be a noninfectious etiology. Donor next of kin reported that no donors had had symptoms of COVID-19 or contact with persons known to have COVID-19. One donor was screened for SARS-CoV-2 infection by the OPO using a NAT before organ procurement and tested negative.

**Table 1:** Epidemiologic and clinical characteristics of solid organ donors associated with potential SARS-CoV-2 transmission investigations, United States, March–May 2020*

Donor	Cause of death	Organs procured from donor and transplanted into other recipients	Chest radiograph and chest CT findings	Donor lung disposition	Results of BAL PCR	Results of serum PCR	Results of nasopharyngeal PCR
A	Hemorrhagic stroke	Bilateral lungs, liver, left kidney	Bilateral lower lobe consolidations	Both lungs transplanted	Negative	Negative	NT
B	Ischemic stroke	Right lung, liver, left kidney	Bilateral lower lobe consolidations	Single lung not allocated in time	NT	Negative	NT
C	Opioid overdose	Bilateral lungs, liver, left kidney, right kidney, pancreas	Bilateral lower lobe consolidations	Both lungs transplanted	NT	Negative	NT
D	Head trauma	Liver, left kidney, right kidney, heart	Bilateral lower lobe consolidations	Lungs not transplanted because of traumatic damage	NT	Negative	NT
E	Hemorrhagic stroke	Bilateral lungs, right kidney, left kidney/split liver, split liver, heart	No focal infiltrates, small pneumomediastinum	Both lungs transplanted	NT	Negative	NT
F	Head trauma	Left lung, right lung, liver, and heart	Bilateral lower lobe consolidations	Both lungs transplanted	NT	Negative	NT
G	Head trauma	Heart/left kidney, liver, right kidney/pancreas	Bilateral lower lobe consolidations	Lungs not transplanted because of abnormal chest imaging	NT	Negative	NT
H	Opioid overdose	Heart, left kidney, right kidney, liver	Patchy ground glass in all lobes	Lungs not transplanted because of abnormal chest imaging	NT	Negative	Negative

*In the 14 days before death, none of the donors had known contact with someone who had been sick with or received a diagnosis of coronavirus disease, had traveled, or had reported nosocomial transmission of SARS-CoV-2 in the donor hospital. None of the donors experienced symptoms consistent with COVID-19, including fever, cough, and shortness of breath. BAL, bronchoalveolar lavage; CT, computed tompgraphy; COVID-19: coronavirus disease; NT, not tested; SARS-CoV-2, severe acute respiratory syndrome coronavirus 2.

Among the 8 index recipients, 4 received lung, 2 received liver, and 2 received heart transplants (Table 2). The median age of index recipients was 65 years (range 37–75 years); the median duration from organ transplantation to symptom onset was 9 days (range 6–81 days). Seven (88%) index recipients experienced fever or lower respiratory tract symptoms. Seven index recipients required mechanical ventilation; 3 of them (2 liver recipients and 1 lung recipient) died. All index recipients had potential or confirmed community or healthcare exposure to persons infected with SARS-CoV-2. 

**Table 2:** Epidemiologic and clinical characteristics of index recipients associated with SARS-CoV-2 transmission in recipients of solid-organ transplants, United States, March–May 2020*

Index recipient	Donor	Organ	Symptoms	No. days from transplant to symptom onset	Exposure to persons with COVID-19	No. days in hospital†	No. days in ICU‡	No. days intubated‡	COVID-19 treatment	Immunosuppressive medications, induction	Immunosuppressive medications, maintenance	Patient status
A	A	Bilateral lungs	Fever, respiratory failure, diaphoresis, atrial fibrillation, shock, acute kidney failure	6	Confirmed COVID-19 among HCW contacts, potential nosocomial transmission	109	35	41	Hydroxychloroquine, azithromycin	Basiliximab, methylprednisolone	Mycophenolate, tacrolimus, prednisone	Alive
B	B	Single lung	Fever, nasal congestion	14	Confirmed COVID-19 among HCW contacts	31	None	None	Hydroxychloroquine	Basiliximab, methylprednisolone	Mycophenolate, tacrolimus, prednisone	Alive
C	C	Liver	Altered mental status, fever, respiratory failure	9	Confirmed COVID-19 among household contacts	79	66	64	Convalescent plasma	Basiliximab	Everolimus, prednisone	Dead
D	D	Liver	Cough	7	Suspected nosocomial transmission at transplant center	49	19	19	Hydroxychloroquine, azithromycin	Methylprednisolone	Tacrolimus, mycophenolate mofetil, prednisone	Dead
E	E	Bilateral lungs	None	Asymptomatic	Confirmed COVID-19 among HCW contacts	17	4	2	Chloroquine	Basiliximab, methylprednisolone, mycophenolate mofetil	Tacrolimus, mycophenolate mofetil, prednisone	Alive
F	F	Single lung	Fever, dyspnea, wheezing	7	Suspected nosocomial transmission at transplant center	14	14	9	Remdesivir, azithromycin, intravenous immunoglobulin	Basiliximab	Tacrolimus, mycophenolate mofetil, prednisone	Dead
G	G	Heart and single kidney	Tachycardia, respiratory failure	81	Confirmed COVID-19 among household contacts	10	9	7	Hydroxychloroquine, azithromycin	Basiliximab, methylprednisolone	Tacrolimus, mycophenolate mofetil, prednisone	Alive
H	H	Heart	Dyspnea, fever, diarrhea	9	Suspected nosocomial transmission at transplant center	84	77	9	Remdesivir, azithromycin, tocilizumab	Basiliximab, methylprednisolone, mycophenolate mofetil	Tacrolimus, mycophenolate mofetil, methylprednisolone	Alive

*For all recipients, SARS-COV-2 test was by nasopharyngeal swab. COVID-19, coronavirus disease; HCW, healthcare worker; ICU, intensive care unit; SARS-COV-2, severe acute respiratory syndrome coronavirus 2. †If patient never discharged since organ transplantation, number of days hospitalized since organ transplantation. If patient discharged and re-admitted, number of days hospitalized since most recent admission. ‡For patients C, D, and F, day of death is considered last day of hospitalization, day in ICU, or day intubated.

Organs from the 8 deceased donors were transplanted into 31 recipients, including the 8 index recipients. Of the 23 co-recipients, 11 (48%) were tested for SARS-CoV-2 infection using a NAT; 1 tested positive 41 days after transplant. Twelve co-recipients were not tested because of absence of symptoms and need to conserve test supplies. Within 14 days after transplant, 3 co-recipients manifested symptoms related to COVID-19, but all tested negative.

## Conclusions

The 8 potential donor-derived SARS-CoV-2 transmissions reported to the OPTN during March–May 2020 were referred to CDC for public health investigation. Although the source of transmission was not definitively established, the available evidence did not suggest transmission occurred from donors.

The risk for organ donor-derived SARS-CoV-2 transmission is unknown (*1*,*3*). Transmission of severe acute respiratory syndrome coronavirus, Middle East respiratory syndrome coronavirus, or SARS-CoV-2 from an organ or blood donor to a recipient has not been reported as of November 2020 (*1*). However, recent studies documented the presence of viral particles in organs of patients who had severe COVID-19 or died from COVID-19 (*4*–*6*). Infectious SARS-CoV-2 has been isolated from respiratory specimens, stool (*7*), and urine (*8*), suggesting transmissible virus might be present in extrapulmonary organs. Although these studies suggest that transplant transmission is plausible, the risk for SARS-CoV-2 transmission from extrapulmonary organs of asymptomatic infected deceased donors to organ recipients is unknown. Evidence suggests that the risk for viremia in persons with asymptomatic COVID-19 is low (*9*). However, OPOs should continue to evaluate donors for evidence of SARS-CoV-2 infection (*10*) because transmission of SARS-CoV-2 from organ donor to recipient might be possible and subsequent recipient infection might be severe; evaluating donors could also protect organ procurement and transplantation clinical teams. The American Society of Transplantation has recommended testing all donors by NAT since May 2020. No donors in this study had reported contact with persons with confirmed or suspected COVID-19.

COVID-19 has an estimated incubation period of 2–14 days (10), and all index recipients had confirmed or potential SARS-CoV-2 exposure during the 14 days before symptom onset or diagnosis. No co-recipients contracted COVID-19 within 14 days of transplant, providing further support that the donor was not the source of transmission. Transplant recipients and their healthcare providers should continue to take steps to reduce SARS-CoV-2 exposure.

Of the 8 index recipients in this study, 7 were intubated and 3 died. Seven of the index recipients received their COVID-19 diagnosis within 14 days of transplantation, which suggests that recipients of recent transplants may be at increased risk for severe disease compared with the general population (*11*) and possibly with organ recipients whose transplants were done months or years before SARS-CoV-2 infection (*12*). The advanced age of the index recipients in our study might have contributed to increased illness. Although some COVID-19 case series have suggested that organ transplant recipients are at higher risk for severe disease than the general population, others suggest that disease severity is similar (*2**,**11*). Data are sparse on the clinical severity of COVID-19 in recently transplanted organ recipients. 

This study is subject to the following limitations. First, 7 of 8 donors were not tested for SARS-CoV-2 before transplant, and stored respiratory specimens were unavailable for retrospective testing. Although donor serum specimens were tested by NAT, limited performance and sensitivity data are available for this sample type using this test, and SARS-CoV-2 viremia is likely uncommon and intermittent (*1*). Second, donors and recipients might have had contact with unidentified persons with SARS-CoV-2 infection, including asymptomatic or presymptomatic persons (*13*). Asymptomatic SARS-CoV-2 infection might not have been detected in co-recipients given the low rate of testing (<50%). Finally, donor-derived SARS-CoV-2 transmission might not have been recognized by transplant clinicians and therefore not reported for investigation.

COVID-19 in the organ transplant recipients we report appears to have been community- or hospital-acquired. These findings suggest that organ transplant recipients, particularly in the immediate posttransplant period, might be at increased risk for severe COVID-19. Measures to limit household and healthcare-associated SARS-CoV-2 transmission to recipients should be implemented (*10**,**14**,**15*). All suspected donor-derived SARS-CoV-2 infections should be reported to the OPTN for further investigation.

Appendix Additional information about investigation of SARS-CoV-2 infections among recent organ recipients.
