# Supplementary material for: SARS-CoV-2 Infections among Recent Organ Recipients, March–May 2020, United States
Source: Emerg Infect Dis. 2021 Feb;27(2):552–5. doi: 10.3201/eid2702.204046 (PMC7853574; doi:10.3201/eid2702.204046)
Supplement: Appendix — Additional information about investigation of SARS-CoV-2 infections among recent organ recipients. [file 20-4046-Techapp-s1.pdf]

Article DOI: https://doi.org/10.3201/eid2702.204046

SARS-CoV-2 Infections among Recent Organ Recipients, March–May 2020, United States

Appendix

Index Recipient Investigation

Methods

All organ procurement organizations (OPOs) and transplant centers in the United States are members of the Organ Procurement and Transplantation Network (OPTN). Current OPTN policy requires transplant centers and OPOs to report unanticipated potential donor-derived transmission events to the OPTN for investigation by DTAC. CDC coordinates investigations involving pathogens of special interest, including severe acute respiratory syndrome coronavirus 2 (SARS-CoV-2).

For all reported potential donor-derived SARS-CoV-2 transmissions, CDC, OPO, or transplant center staff reviewed medical records of donors and recipients, including SARS-CoV-2 testing and chest imaging reports. The donor clinical history was reviewed to look for evidence of symptoms consistent with COVID-19, including fever, cough, or shortness of breath. OPO staff asked donor next of kin if the donor had been exposed to someone who had been diagnosed with COVID-19. Additional information included whether the donor had contact with any sick persons or any travel in the 2 weeks prior to death. Recipients who initially tested positive for SARS-CoV-2 infection and triggered a notification to the OPTN of a potential donor-derived transmission were referred to as index recipients, and recipients who shared a common donor with index recipients are referred to as co-recipients. CDC investigators asked OPO and index recipient hospital staff if nosocomial transmission of SARS-CoV-2 had occurred in donor or recipient hospitals. CDC investigators collected information from transplant centers and OPOs about potential exposure to SARS-CoV-2 for index recipients during the 14 days before symptom onset, including contact with persons with fever or respiratory symptoms or a diagnosis

of COVID-19, travel, and hospitalization prior to admission for the transplant procedure. Confirmed exposure was defined as reported contact (e.g., living with a person or being treated by a healthcare worker) with a person diagnosed with COVID-19 during the infectious period (*1*). Potential healthcare exposure was defined as treatment in a healthcare facility with known or suspected nosocomial transmission without a confirmed contact. Potential community exposure was defined as contact with a person meeting a probable COVID-19 case definition (*2*).

Transplant hospital providers monitored organ recipients for symptoms of COVID-19 (fever, cough, shortness of breath, fatigue, myalgias, sore throat, headache, congestion, and other concerning symptoms) for ≥14 days following the transplant. Some asymptomatic recipients, depending on hospital capacity, and recipients who developed symptoms were tested for SARS-CoV-2 infection using a nucleic acid test (NAT) on a nasopharyngeal specimen.

Because donor respiratory specimens (e.g., sputum, bronchioalveolar lavage, nasopharyngeal swab) were only available for 1 donor, donor serum (which is archived per routine OPO practice) was tested for the presence of SARS-CoV-2 RNA by processing with the EZ1 nucleic acid extractor with the EZ1 DSP Virus Kit (QIAGEN, https://www.qiagen.com) followed by real-time RT-PCR as previously described (*3*,*4*).

Results

Donor Clinical and Epidemiologic Findings

For all donors included in this study, the cause of death was determined to be a noninfectious etiology, including stroke, opioid overdose, and head trauma. Next of kin of the 8 organ donors reported that none of the donors had symptoms of COVID-19 or contact with persons known to have COVID-19. Donor chest imaging reports documented infiltrates in 7 (88%) donors. For 4 of those 7 (57%) donors, OPO or the lung transplant center medical staff determined the chest imaging results to be unremarkable, and the lungs were transplanted. Lungs from 1 donor had evidence of trauma and were not transplanted. Due to abnormal chest imaging, lungs from 2 donors were not transplanted. One donor was screened for SARS-CoV-2 infection using a NAT on a respiratory specimen by the OPO prior to organ procurement and tested negative; the test was conducted using an in-house assay authorized by the US Food and Drug Administration under an Emergency Use Authorization. None of the 8 donor serum specimens had detectable SARS-CoV-2 RNA. No donors were tested for SARS-CoV-2 antibodies.

Index Recipients

Of the 8 index recipients, 4 received lung, 2 received liver, and 2 received heart transplants (Table 2). Seven (88%) index recipients were male, and the median age of index recipients was 65 years (range 37–75 years); the median duration from organ transplantation to symptom onset was 9 days (range 6–81 days). Seven (88%) index recipients developed fever or lower respiratory tract symptoms. Seven index recipients required mechanical ventilation; 3 of those, 2 liver recipients and 1 lung recipient, died. Seven index recipients were given induction immunosuppressive therapy that included basiliximab, an interleukin-2 antagonist. All index recipients received investigational therapy for COVID-19. Treatment regimens included hydroxychloroquine, azithromycin, remdesivir, convalescent plasma, intravenous immunoglobulin, or tocilizumab. All index recipients had potential or confirmed community or healthcare exposure to persons infected with SARS-CoV-2. Four (50%) index recipients had close contact with a healthcare worker with laboratory-confirmed SARS-CoV-2 infection during the 14 days prior to symptom onset. Two index recipients were hospitalized in facilities with potential nosocomial transmission. Two index recipients had contact with household members with laboratory-confirmed COVID-19. Only 1 index recipient had a pretransplant specimen available for testing, and it tested negative by NAT for SARS-CoV-2. Detailed clinical reports of index recipients A and G have been published (*5*,*6*).

Co-recipients

From the 8 deceased donors, organs were transplanted into 31 recipients, including the 8 index recipients (Appendix Table). Among the 23 co-recipients, 11 (48%) were tested for evidence of SARS-CoV-2 infection; all tests used a NAT on a nasopharyngeal swab or bronchoalveolar lavage specimen. Ten of these 11 co-recipients tested negative. One asymptomatic co-recipient was tested 41 days after transplant when she was admitted for a routine follow-up procedure and tested positive. None were tested for SARS-CoV-2 antibodies. Within 14 days after transplant, 1 co-recipient experienced fever, 1 required additional oxygen, and 1 had diarrhea. All 3 of these recipients tested negative for SARS-CoV-2 infection. Three co-recipients experienced symptoms, including vomiting, diarrhea, shortness of breath, or rhinorrhea, $\leq$14 days after transplant, but all tested negative for SARS-CoV-2 infection. SARS-CoV-2 testing was not performed on the other 12 co-recipients because these patients did not

have COVID-19 symptoms, and the transplant centers prioritized testing capacity for symptomatic patients. No co-recipients were tested for SARS-CoV-2 antibodies.

References

1. Centers for Disease Control and Prevention. Operational considerations for adapting a contact tracing program to respond to the COVID-19 pandemic. 2020 [cited 2020 October 27]. https://www.cdc.gov/coronavirus/2019-ncov/global-covid-19/operational-considerations-contact-tracing.html

2. Centers for Disease Control and Prevention. Coronavirus disease 2019 (COVID-19) 2020 interim case definition. 2020 [cited 2020 October 27]. https://wwwn.cdc.gov/nndss/conditions/coronavirus-disease-2019-covid-19/case-definition/2020/08/05

3. Lu X, Wang L, Sakthivel SK, Whitaker B, Murray J, Kamili S, et al. US CDC real-time reverse transcription PCR panel for detection of severe acute respiratory syndrome coronavirus 2. Emerg Infect Dis. 2020;26:1654–65. PubMed https://doi.org/10.3201/eid2608.201246

4. US Department of Health and Human Services. Protection of human subjects, 45 C.F.R. Section 46 (2018).

5. Keller BC, Le A, Sobhanie M, Colburn N, Burcham P, Rosenheck J, et al. Early COVID-19 infection after lung transplantation. Am J Transplant. 2020;20:2923–7. PubMed https://doi.org/10.1111/ajt.16097

6. Vilaro J, Al-Ani M, Manjarres DG, Lascano JE, Cherabuddi K, Elgendy AY, et al. Severe COVID-19 after recent heart transplantation complicated by allograft dysfunction. JACC: Case Reports. 2020;2:1347–50. https://doi.org/10.1016/j.jaccas.2020.05.066

Appendix Table. COVID-19 symptoms and SARS-CoV-2 test results of non-index solid organ recipients (co-recipients) associated with potential SARS-CoV-2 transmission investigations, United States, March–May 2020

Donor	Organ received	Symptoms of COVID-19 (days posttransplant at which symptoms started)	SARS-CoV-2 PCR results (days posttransplant at which tests performed)
A	Liver	None	Not tested
	Left kidney	None	Not tested
B	Liver	None	Negative nasopharyngeal swab (18)
	Left kidney	None	Negative nasopharyngeal swab (18)
C	Bilateral lungs	None	Negative bronchoalveolar lavage specimen (17)
	Left kidney	None	Negative nasopharyngeal swab (17)
	Right kidney	Fever (14)	Negative nasopharyngeal swab (18)
	Pancreas	Rhinorrhea (26)	Negative nasopharyngeal swab (26)
D	Left kidney	None	Not tested
	Right kidney	None	Not tested
	Heart	None	Positive nasopharyngeal swab (41)
E	Right kidney	None	Not tested
	Left kidney/split liver	None	Not tested
	Split liver	None	Not tested
	Heart	None	Not tested
F	Right lung	Hypoxia (8)	Negative nasopharyngeal swab (8, 9, 12)
	Liver	Diarrhea and vomiting (15)	Negative nasopharyngeal swab (18, 20)
	Heart	Diarrhea (10)	Negative nasopharyngeal swab (7, 10)
G	Liver	None	Not tested
	Right kidney/pancreas	None	Not tested
H	Left kidney	None	Not tested
	Right kidney	None	Not tested
	Liver	None	Negative nasopharyngeal swab (19, 26)
